# Development of optimisation methods to identify sources of pollution and assess potential health risks in the vicinity of antimony mines

**DOI:** 10.1007/s10653-025-02369-0

**Published:** 2025-02-11

**Authors:** Dragan Čakmak, Veljko Perović, Dragana Pavlović, Marija Matić, Darko Jakšić, Samat Tanirbergenov, Pavle Pavlović

**Affiliations:** 1 Department of Ecology, Institute for Biological Research ‘Siniša Stanković’, University of Belgrade, Bulevar Despota Stefana 142, 11108 Belgrade, Serbia; 2 Institute of Agricultural Economics, Volgina 15, 11060 Belgrade, Serbia; 3 Department of Agrochemistry and Soil Ecology, U.Uspanov Kazakh Research Institute of Soil Science and Agrochemistry, 75 Al-Farabi Ave, 050060 Almaty, Kazakhstan

**Keywords:** Potential toxic elements, Network analysis, Compositional data analysis, Receptor models, Health risk factorisation

## Abstract

After elevated levels of lead (Pb) were found in the blood of children living near the antimony (Sb) mine and battery smelter in Zajača, Republic Serbia, studies were carried out to determine the health risk assessment (HRA) effects of the soil. In this study, for the first time a combination of Network Analysis, CoDA (Compositional Data Analysis) and receptor modelling was used to determine the geopedological and atmospheric origin of PTEs in soil and their historical significance. It was found that arsenic (As) and Pb are the main pollutants in the area. The largest contribution to the environmental risk (Er) was made by Pb. In addition to perception methods Network Analysis (NA) was used to determine the source of pollution and, for the first time, the strength of the positive and negative connections of the network nodes of the mutual influence of PTE. Lead pollution was found to originate from two sources: historical and present, and As was found to originate from a wider area. For the child population, an unacceptable risk for the occurrence of chronic diseases with a probability of 95% was found, with As and Pb accounting for the highest percentage. Similarly, As has the greatest impact on occurrence of cancer at the unacceptable risk level, while Pb is at the notable risk level. The historical exposure to Pb is slightly lower and the difference is slightly more pronounced for total pollution for HRA.

**Supplementary Information:**

The online version contains supplementary material available at 10.1007/s10653-025-02369-0.

## Introduction

The capacity of the soil to ensure the development of the ecosystem can be expressed through its ecological and socio-economic functions, between which there are numerous contradictions, which is particularly pronounced in the case of land use. The quality and health of the soil is of great importance for the quality of the environment, which is particularly pronounced in industrial areas as a result of the necessary economic development and its inevitable impact on the ecological balance of the environment (Belanović Simić et al., 2023; Wu et al., 2011). Since industrial areas are usually built close to settlements with a larger population in order to reduce the cost of transporting labour, their impact complicates the problem further. One of the aspects of the harmful environmental impact of industrial areas is the emission of harmful trace elements, regardless of whether they are the target of a particular industry or the result of technological processes (Belanović Simić et al., 2023; Pavlović et al., 2018; Saljnikov et al., 2019).

Potentially toxic elements (PTEs) are one of the main pollutants of the ecosystem, including soil, due to their strong binding to soil particles, which makes them difficult to remove (Cakmak et al., 2018; Sastre et al., 2002). The main source of potentially toxic elements (PTE) is the geological substrate (Čakmak et al., 2020; Mcllwaine et al., 2017), while their amount increases significantly in soils of industrial areas and their impact becomes more complex due to anthropogenic influence (Islam et al., 2015; Mihailović et al., 2015). Further reasons for the difficulty in determining the effects on human health in industrial areas are certainly the high population density in a small area and the different modes of action of the harmful trace elements (Sieghardt et al., 2005). Children are the most exposed due to their natural sensitivity (Granero & Domingo, 2002; Mielke et al., 1999). For all these reasons, the systematic and qualitative determination of the effects of harmful microelements in industrial areas near industrial centres is very difficult (Wu et al., 2011).

One of the most reliable methods to determine harmful effects of certain microelements is the detection of certain diseases and elevated levels of certain elements in the tissues and blood of the exposed population. Once the target microelements are identified as objects of research, the specific sources of their distribution and their potentially harmful future effects on the exposed population must be determined, taking into account the environment in which they are deposited.

One of the most threatened industrial areas in Serbia, but also in the Western Balkans, is the settlement of Zajača. Mining activities in this area were recorded as early as 1445, and the current lead and antimony mine was opened in 1887, while ore processing centre was first established in Zajača in 1896, becoming a battery recycling centre in 1980 (IKS, 2012; MEP, 2016). Research on the children population in this settlement revealed elevated blood lead levels (BLL) three times higher than the limit value (Mandić-Rajčević et al., 2018; Centers for Disease Control, Centers for Disease Control, 2022; Council, 1993). Considering the location and previous research, As, Pb and Sb are particularly emphasised as pollutants that have a major impact on human health. Long-term As exposure can influence the occurrence of skin, lung and bladder cancer (Hong et al., 2014), Pb exposure leads to chronic lung diseases, lung cancer and disorders of the nervous, cardiovascular and digestive systems as well as kidney function (Boskabady et al., 2018). Sb, on the other hand, leads to disorders of the cardiovascular system, the nervous system, liver damage and causes conjunctivitis and dermatitis. It also has an effect on mutagenicity (Muhammad Shahid et al., 2019).

In this study, for the first time a combination of Network Analysis, CoDA (Compositional Data Analysis) and receptor modelling was used to determine the geopedological and atmospheric origin of PTEs in soil and their historical significance. Thus, Network analysis (NA), to determine the strength of positive and negative connections of network nodes of individual PTEs in the soil, is a mathematical-statistical method based on the structural linking of individual independent data sets and the determination of the strength and type of their connections (Matić et al., 2023). Compositional Data Analysis (CoDA) is a receptor method (Buccianti et al., 2008) that is well suited for grouping specific PTEs to determine fundamental geochemical processes thanks to the ilr transformation into basic geochemical processes. It is widely used to determine the source and influence of certain elements in areas near mines (Tepanosyan et al., 2020). Principal component analysis (PCA) is one of the factor methods used to determine the sources of individual PTEs from large environmental monitoring databases (Ali et al., 2016). The positive matrix factorisation (PMF) model is a receptor method, and it is also a widely used method for determining the source of individual PTEs, it is primarily used to determine the sources of PTEs in dynamic systems such as the atmosphere and sediment (Čakmak et al., 2023; Zhi et al., 2016). The Health Risk Assessment (HRA) method (USDOE 2011; USEPA 1989) makes it possible to determine, based on the total amount of PTEs in the soil, their effects and the probability of occurrence of chronic diseases in children and adults, as well as the probability of cancer occurrence in such an environment. However, realistically obtained data often cannot fully reflect the actual situation of the impact of PTEs on human health. The ability to predict the impact of PTEs on public health through soil can be made more efficient by using the Monte Carlo simulation (MSC) and determining the percentage probability of such occurrence (Jin & Lv, 2021).

With this in mind, an integrated methodological approach was implemented to obtain answers to the following important questions:

1. What is the environmental hazard posed by As, Pb and Sb in the study area and how great is it?
2. What are the individual sources that contribute most to soil pollution and is it possible to determine their historical contribution?
3. What is the risk level to the resident population, especially children?

## Materials and methods

### Study area and sampling

**Fig. 1:**
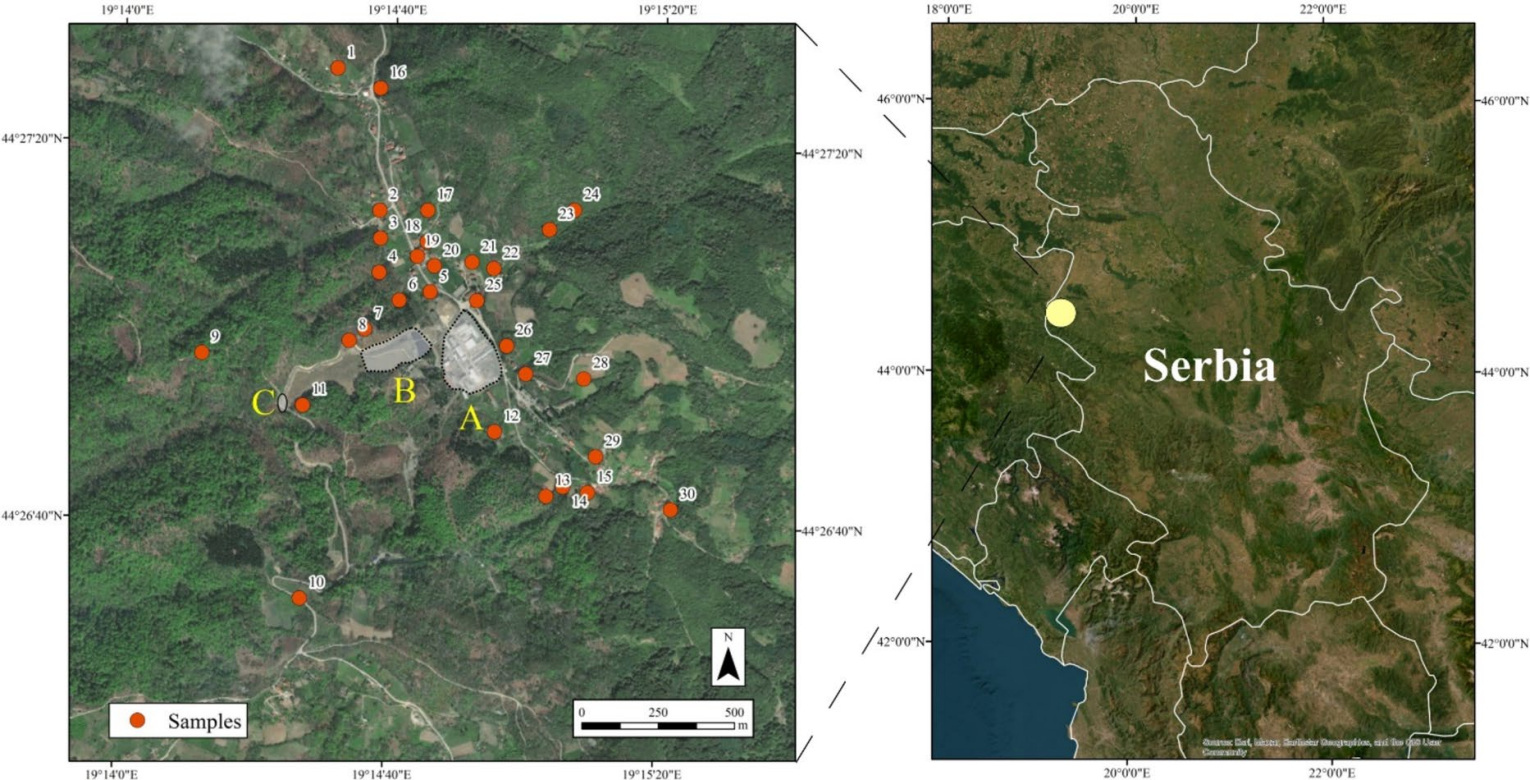
Study area and sampling locations (A-location of the smelter; B-location of tailings; C-entrance to the mine)

The samples were taken 2016 in the vicinity of the Zajača settlement (between 190 14′20″E–′190 15′40 ″E 190 14′20″E–′190 15′40″E and 440 26′55″N–440 27′50N), located 12 km from the regional centre of Loznica. Zajača has a population of 576 inhabitants. The nature of the location determined the method of sampling, i.e. the inclusion of the Štira River basin with peak points, which should give a complete picture of the forms of pollution and its distribution. It should be noted that the left bank of the river differs significantly in terms of the form and type of pollution, as the incision caused by the river is due to the geological substrate, and on this left side, i.e. southwest of the village of Zajača, ore deposits have been created, the first of which is located in the immediate vicinity of the antimony mine (MEP, 2016). As part of the investigation, 30 soil samples were taken from the depth of up to 10 cm. Each sample represents a composite sample of 5 individual samples taken according to the “LUCAS” methodology. This means that four samples were taken at a distance of 2 m from the central individual sample and aligned according to the cardinal points (LUCAS 2012). The individual positions of the composite samples were determined using a GPS device (Fig. 1). After air-drying, they were ground to 2 mm in a steel mill. A chemical analysis was then carried out.

### Soil samples analysis

Total carbon was determined using a CHN analyzer (Vario EL III-Elementar Analysensystem GmbH, Hanau, Germany) by dry combustion at 1150 °C (Nelson & Sommers, 1996). The Scheibler calcimeter method was used to determine the CaCO_3_ content, while the organic carbon (C organic) content was calculated as the difference between the total and inorganic carbon. The granulometric composition was determined using the pipette method with the International B modification (Gee & Or, 2002). The following analyses were carried out: The hot acid extractable content of potentially toxic elements (PTEs) (As, Cd, Co, Cr, Cu, Fe, Mn, Ni, Pb, Zn and Hg) was determined using an ICAP 6300 optical emission spectrometer (Thermo Electron Corporation, Cambridge, UK) after digestion in aqua regia (ISO 11466:^2004^). All chemical analyses were performed on two replicates. To verify the results, the reference soil was analysed for the presence of microelements (reference ERM-CC141 clay soil, Belgium, with a precise concentration of microelements soluble in aqua regia to ensure higher accuracy of the metre). The recovery rate ranged from 89 to 112%. The lower detection limits for the elements were as follows: 0.6436 (As), 0.0166 (Cd), 0.0326 (Co), 0.2897 (Cr), 2.3233 (Cu), 81.4905 (Fe), 0.0172 (Hg), (0.4852 (Mn), 0.2239 (Ni), 2.8597 (Pb), 0.0851 (Sb) and 8.5106 (Zn) mg kg^−1^. The relative standard deviation (RSD) was < 5% for each element.

### Assessment of the degree of soil pollution

Target and intervention values are values that in some way define the pollution from each PTE (SEDL, 2000). The target value is the basic concentration value below which compounds and/or elements are known or suspected to have no effect on the natural properties of the soil (based on the background value). The intervention value (remediation value) is the maximum tolerable concentration above which remediation is required (based on studies by the National Institute for Dutch Public Health and Environmental Protection of both human and ecotoxicological effects of soil contaminants). It is very important to note that when calculating these values, the content of clay and organic matter, which strongly influence the availability or toxicity of certain PTEs, are included in the formula (Adriano, 2002). These values are determined using the following equation:

1 $$ \left( {SW,IW} \right)b = \frac{{\left( {SW,IW} \right)*A + \left( {B*clay} \right) + \left( {C*organic matter} \right)}}{{\left[ {A + \left( {B*25} \right) + \left( {C*10} \right)} \right]}} $$

where: (SW)b-target value (Table S1); (IW)b-intervention value (Table S1); A,B,C-the constant depending on the type of PTEs, is given by the European Union (EU), (SEDL, 2000), (Table S1); clay—% of particle size < 2 μm; Organic matter—% of organic matter.

One of the measures of pollution, i.e., ecological vulnerability under the influence of PTEs in the soil, is the Individual ecological risk (Er), which is calculated according to the following equation:

2 $$ Er = Tr\frac{Cn}{{GB}} $$

where: Tr-toxicity coefficient for the specific PTE, for the following elements it is: As-10; Pb-5, Hakanson, 1980) and Sb-7 (Wang et al., 2018); Cn-concentration of the element; GB- background value of the elment (MEP, 2016); the values of Er are as follows: < 40 low; 40–80 medium; 80–160 significant; 160–320 high; > 320 very high (Hakanson, 1980).

### Determining the source of the individual PTEs

Network analysis (NA) enables the analysis of investigated variables that can form multidirectional relationships that can only be partially revealed by other statistical methods. The complex analysis is presented graphically, with negative relationships shown in red and positive relationships in green, with the thickness of the line indicating significance. The NA thus essentially consists of two basic forms (nodes and edges), with the nodes indicating the mutual formation of groups between different PTEs, while the edges reflect the strength of their relationship. Central nodes in the network are determined by calculating centrality measures. In this study, expected influence (EI) was used to evaluate all nodes in the network, as it also accounts for negative associations (Robinaugh et al., 2016). Of course, it should be noted that the EI reflects the sum of the absolute weights of the edges that they share with other nodes in the network (Robinaugh et al., 2016).

Compositional Data Analysis (CoDA) clr-biplot is used with multivariate datasets to describe and visualise relationships between variables expressed as points and rays (Kempton, 1984). Here, the clr-biplot methodology, adapted to the case of compositional data (Aitchison & Greenacre, 2002; Otero et al., 2005), was applied to analyse geochemical variables (Thiombane et al., 2018; RGSCoDa, 2017; Liu et al., 2016). The ray lengths are directly proportional to the clr variance of the corresponding chemical element. The multidimensional representation mainly indicates the common origin of certain elements (Aitchison & Greenacre, 2002). Accordingly, the orthogonality of the compounds also indicates a log-ratio uncorrelation (Thiombane et al., 2018). The clr biplot was created with the software CoDa Pack (CoDaPack-Version 2.03.01, 2017).

Principal component analysis (PCA) as a receptor method was performed to determine the source of each PTE, applying the Varimax rotation matrix to improve the interpretability of the components. The Kaiser criterion (eigenvalue > 1) was used to determine the number of components. The method was carried out using the SPSS 25 programme. The component impact maps were created with the help of ArcGIS software using factor scores.

Positive matrix factorisation (PMF) was used to better define the sources of each PTE, taking into account that their origin in the studied area is due to atmospheric deposition. This technique includes realistic errors in its calculations and has no negative values. The model is represented by the following equation:

3 $$ X = GF + E $$

where: X nmx (m measured chemical species in n samples; G-matrix nxp (proportion of sources in the samples); F-matrix pxm (source composition, source profile). G and F are the factor matrices to be determined, while E represents the residual matrix, the difference between the X- matrix and the model Y = GF as a function of G and F (EPA PMF v. 5.0).

In this study, for data with a detection limit (MDL), the uncertainty matrix (μij) was determined according to the following equations:

4 $$\mu {\text{ij}} = \sqrt {\left( {\sigma  \times {\text{concentration}}} \right)^{2}  + \left( {0.5 \times MDL} \right)^{2} } $$

If MDL ≤ μij then:

5 $$ \mu {\text{ij}} = \frac{5}{6} \times {\text{MDL}} $$

wherein: MDL – method detection limit; σ – the relative sdandard deviation.

In addition, ArcGiIS software was used to create factor influence maps based on the factor scores.

### Determination of the health risk (HRA)

The assessment of the impact of PTEs in soil on health risk was made according to the following equations:

Non-cancinogenic risk for ingestion (Eq. 6), for dermal (Eq. 7) and for inhalation (Eq. 8):

6 $$ HQing = \left[ {\left( {C \times IRS \times RBA \times EF \times ED} \right)/\left( {BW \times AT \times RfDo} \right)} \right] \times 10^{ - 6} $$

7 $$ HQder = \left[ {\left( {C \times SA \times AF \times ABSd \times EF \times ED} \right)/\left( {BW \times AT \times RfDo \times GIABS} \right)} \right] \times 10^{ - 6} $$

8 $$ HQinh = \left[ {\left( {C \times EF \times ED} \right)/\left( {AT \times RfC \times PEF} \right)} \right] $$

where total non-canceroginc risk is calculated:

9 $$ HI = HQing + HQder + HQinh $$

#### Carcinogenic risk

In line with the recommendations listed in (USEPA 2020), the carcinogenic risk for residents is calculated using the following equations:

10 $$ CRing = \left( {\left( {C  \times  IFS  \times  RBA  \times  CSFo} \right){|}AT} \right)  \times  10^{ - 6} $$

11 $$ IFS = \left( {EF  \times  EDa  \times IRSa\left| {BWa} \right.} \right) + \left( {EF  \times  EDc  \times  IRSc\left| {BWc} \right.} \right) $$

12 $$ CRder = \left( {\left( {C  \times  DFS  \times  ABSd  \times  CSFo} \right){|}AT  \times  GIABS} \right)  \times  10^{ - 6} $$

13 $$ DFS = \left( {EF  \times  EDa  \times  SAa\left| {BWa} \right.} \right) + \left( {EF  \times  EDc  \times  SAc  \times  AFc\left| {BWc} \right.} \right) $$

14 $$ CRinh = C  \times  EF  \times  ED  \times  IUR  \times  1000/AT  \times  PEF $$

The total carcinogenic risk (TCR) is the sum of carcinogenic risks for each of the three exposure routes and is calculated using the following equation:

15 $$ TCR = CRing + CRder + CRinh $$

Descriptions and reference values for all parameters in the above equations can be found in Tables S2 and S3 (USEPA, 2020), while the calculations of HQ, HI and CR were performed using USEPA's RSL calculator and user’s guide (EPA; 2024).

The combination of PMF and HRA models was developed to determine the contribution of each factor to the potential occurrence of chronic diseases and cancer in the study area.

MSC was used to determine the probability of health risk for each source. The software was run for 1000 iterations with a probability of 95%.

## Results

### Total content of examined elements and basic pollution indices

Based on the mean values, it can be seen that As pollution is the most pronounced, as its content is above the intervention value, while it is above the target values for Pb and Sb. The highest coefficient of variation (CV) was determined for Sb and somewhat lower for Pb. In contrast to the two previous elements, it is below 100% for As, but its value is very high (Table 1, Table S4).

**Table 1:** Descriptive statistics and indicators of pollution

Elements		As	Pb	Sb
		mg·kg^−1^		
Average		85.11	411.02	3.07
Median		73.53	270.05	1.73
Max		268.48	1987.80	17.47
Min		11.95	43.05	NS
SD		58.16	419.21	4.00
CV %		68.33	101.99	130.29
Critical values		%*		
< Target value		10.00	3.33	36.67
> Target value		23.33	60.00	60.00
> Remediation value		66.67	36.67	3.33
Ecolog. risk	Value	%*		
Low	< 40	60.00	56.67	96.66
Medium	40–80	33.33	26.67	0.00
Significant	80–160	6.67	13.33	3.33
High	160–320	0.00	3.33	0.00
Very high	> 320			

With regard to the critical values, it is noticeable that Pb has the lowest percentage of samples below the target value, i.e. 3.33%, followed by As with 10% and Sb with 36.67%. The largest number of samples belonging to the category above the intervention value was recorded for As with 66.67%, followed by Pb with 36.67%, while the lowest percentage was recorded for Sb with 3.33% (Table 1). The spatial distribution for As shows a wide dispersion of values above the intervention value, i.e. along the Štira riverbed, along the entire settlement and the surrounding area, while exceedance of the target value was found at the edges of these areas. For Pb, narrower areas of exceedance of the intervention value are found in the immediate vicinity of the smelter and the tailings pond, while the distribution of values above the target value for Sb is the same as for As, depending on the location (Fig. S1a, b, c).

With regard to ecological risk, it is noticeable that As and Pb have almost the same percentage of samples that fall in the low (approx. 60%) or medium (approx. 30%) category. For Pb, the percentage in the significant category is the highest at 13.33%, while the high category for the same element is 3.33%. For Sb, 96.66% of the samples were found to fall in the low category (Table 1). Similar to the intervention value, As is spatially more represented in the category than Pb, with Pb posing a significant ecological risk in the vicinity of the smelter and downstream of the tailings pond. Antimony is characterised by medium and significant Er in the area downstream of the tailings pond and the smelter, i.e. where these two influences overlap (Fig. S2a, b, c).

### Determining the source of PTEs

#### Network analysis (NA)

**Fig. 2:**
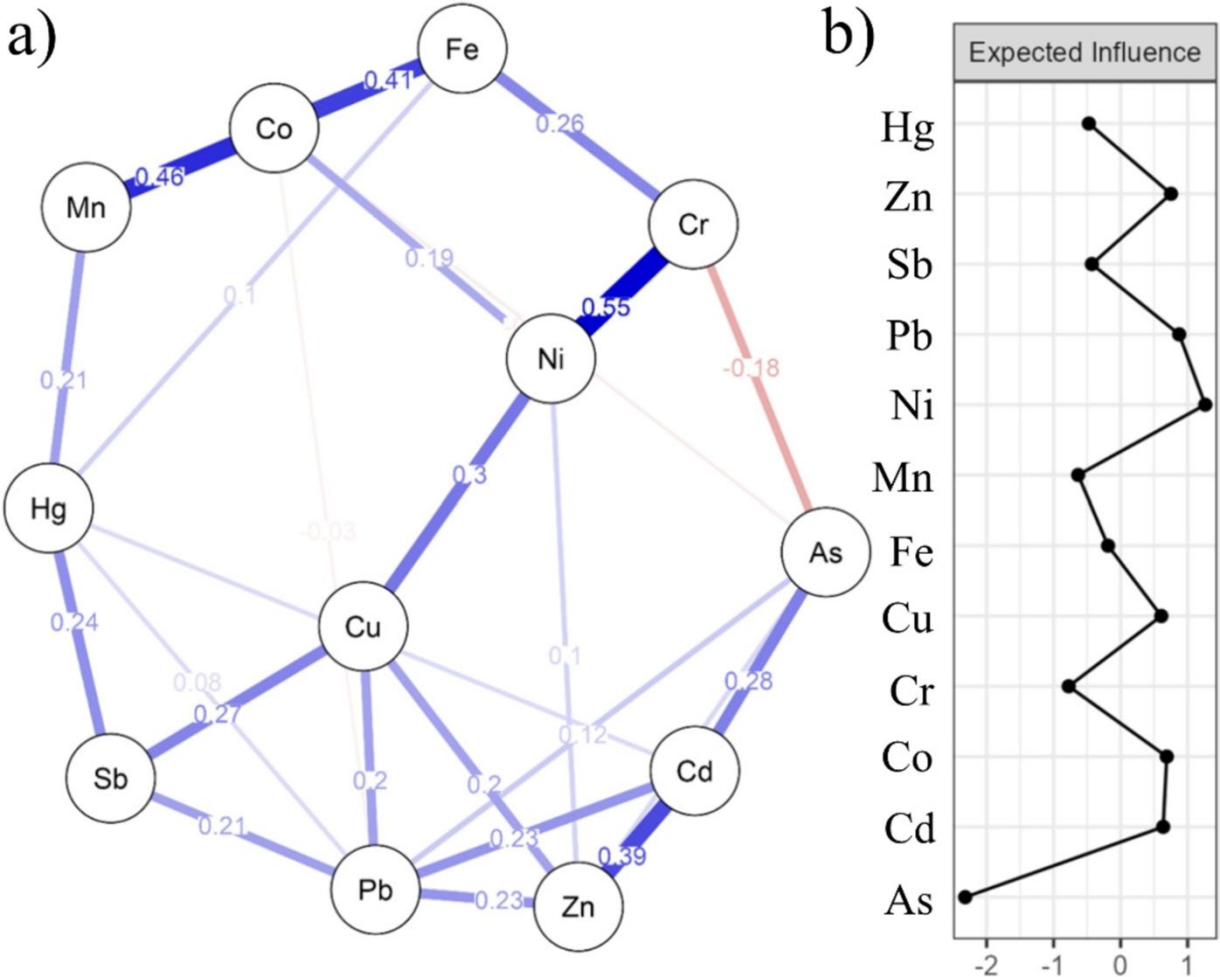
**a** Network analysis (**b**) expected influence of PTEs

There are two nodes in NA. In the first node, Ni stands out, having the highest expected influence value in the entire network (1.263), while in the second node the highest expected influence was determined for Pb (0.874). In the first node, Ni is the most strongly connected to Cr, while it is indirectly connected to Fe and Mn via Co. The first node is directly associated with the second node with Sb via Cu and to a lesser extent with Pb and Zn. Lead is also associated with Hg. A less pronounced connection exists between the first and second nodes: Mn and Hg. Arsenic, which establishes a negative connection with the first node via Cr and Co and also has the lowest expected influence ( − 2.320), stands out clearly in the entire network. In addition to Cd, the binding of this element to Pb is very weak (Fig. 2a, b).

#### Compositional data analysis (CoDA)

**Fig. 3:**
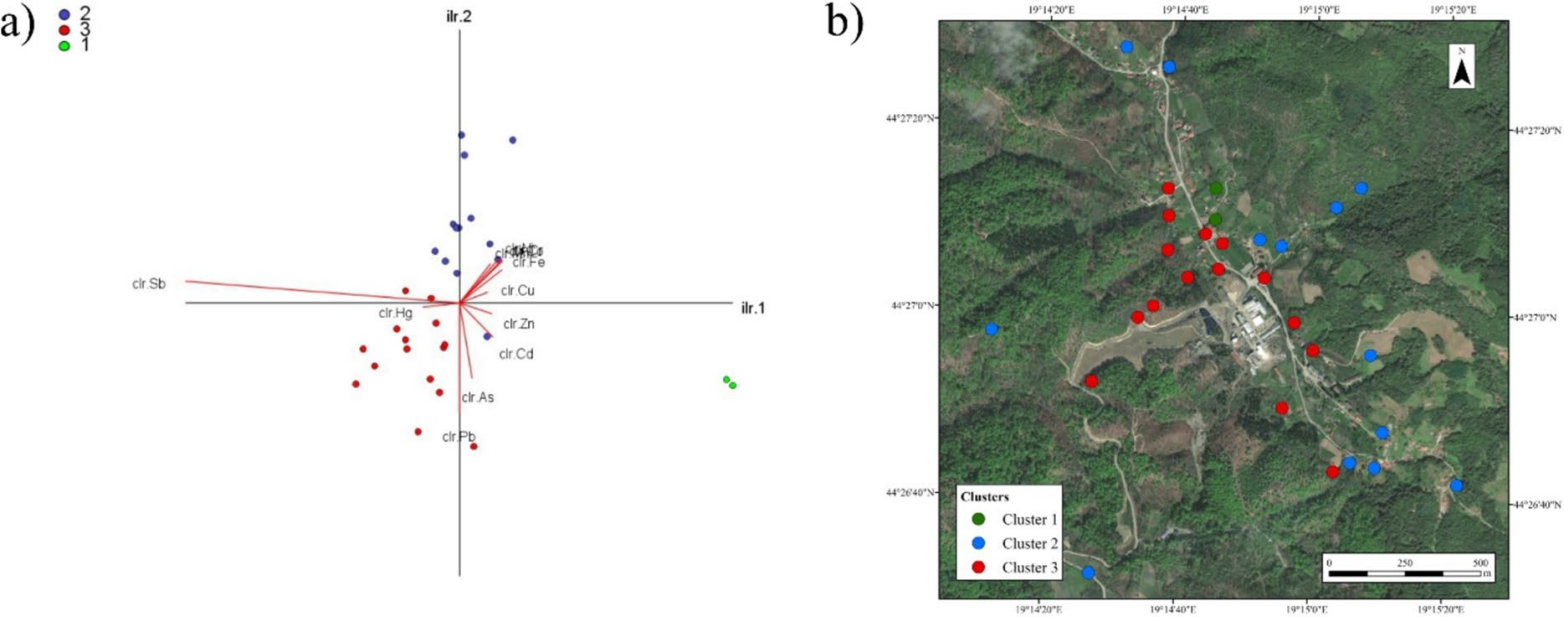
**a** Result of the k-mean clustering on the cir-biplot (**b**) spatial location of the k-means clusters

Three clusters are clearly distinguished in CoDA. Antimony is registered as the element with the strongest geochemical influence in the third cluster, and Hg, Pb and partly As are with it in the same cluster with it. Arsenic is also partly in the first cluster with Cd and Zn. The second cluster represents other elements, although it is clear that the spatial distribution of the clusters is determined by the Štira riverbed. The third cluster is characteristic of the left bank (Fig. 3a, b).

#### Principal component analysis (PCA)

The influence of four factors on the total variance was determined to be 86.38%. The first factor with a share of 28.17% is significantly influenced by As, Cd, Cu, Pb and Zn. The second factor with a share of 22.66% is significantly influenced by Co, Cr, Cu, Fe and Ni. The third factor with a share of 19.75% is influenced by Co, Fe, Mn and Hg. The fourth factor with a share of 15.80% is significantly influenced by Pb, Sb and Hg. (Table S5).

**Fig. 4:**
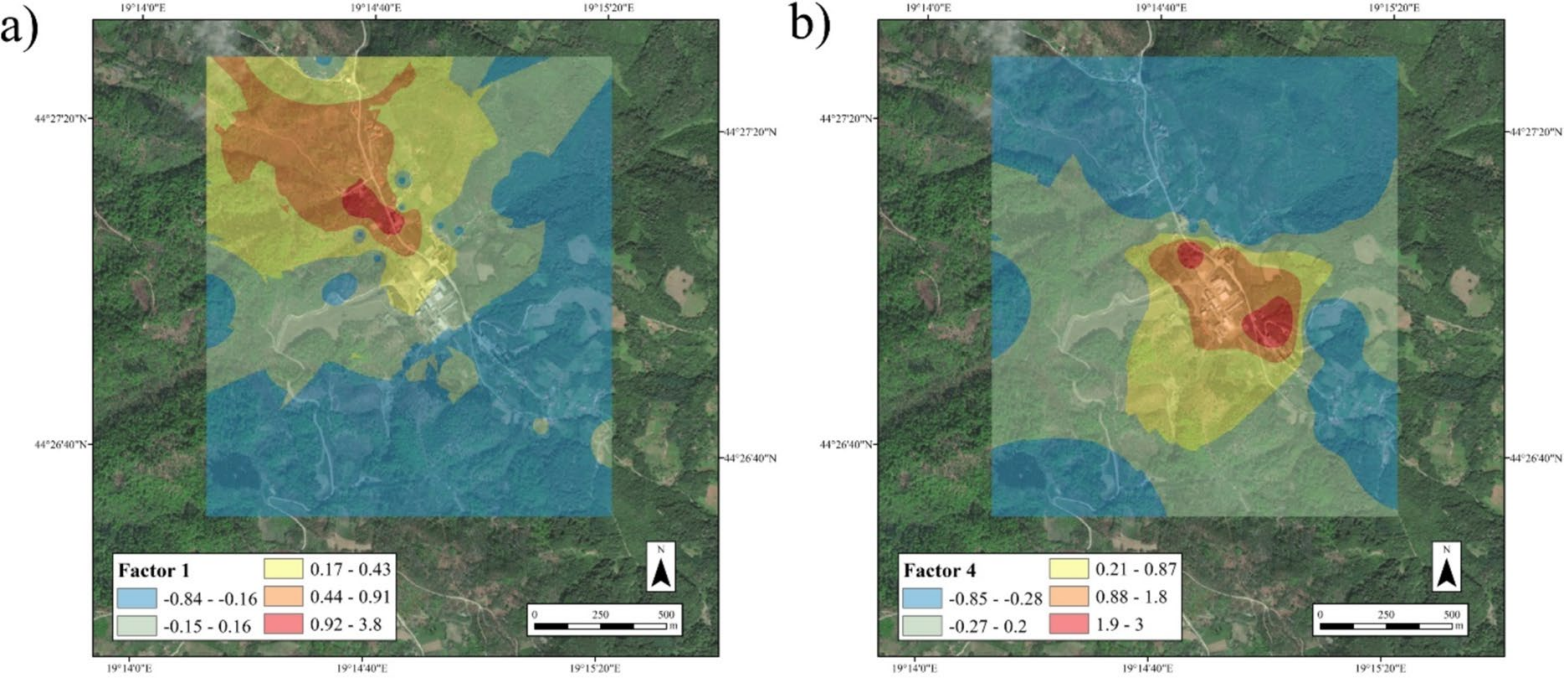
Spatial influence for PCA factors: **a** I factor **b** IV factor

In terms of spatial distribution, the first factor is located downstream of the tailings pond, while the influence of the fourth factor is in the immediate vicinity of the reprocessing plant (Fig. 4 a, b). The second factor extends to both banks of the Štira River, except for the area in the immediate vicinity of the antimony mine dump, and the third factor occupies a large area immediately upstream of the battery recycling plant and the tailings ponds, both of geological origin (Fig. S3 a, b).

#### Positive matrix factorization PMF

The PMF model was set to run 20 times. Residual values between – 3 and 3 were detected in over 85% of the soil samples. The signal-to-noise ratio was strong for all PTE soils when S/N was > 6.2. The R2 values range from 0.60 to 0.99. Based on the previous data that fulfil the basic conditions and the Q value, it was determined that 5 factors are optimal, i.e. one factor more than PCA (Table 2).

**Table 2:** Source composition of PTEs (%) from the PMF model

Species	Factor I	Factor II	Factor III	Factor IV	Factor V
As	59.0	< 0.05	< 0.05	24.8	16.2
Cd	5.7	10.8	26.5	44.9	12.2
Co	12.9	< 0.05	5.2	57	25
Cr	8.0	4.5	36.1	3.9	47.5
Cu	8.6	6.4	37.6	18.7	28.7
Fe	21.8	< 0.05	37.2	< 0.05	41
Mn	13.7	3.2	71.5	10.2	1.4
Ni	< 0.05	4.4	47.5	10.7	37.4
Pb	1.6	38.5	< 0.05	49.8	10.1
Sb	2.4	77.7	< 0.05	< 0.05	19.9
Zn	9.5	< 0.05	35.2	41.9	13.4
Hg	18.5	40.3	36.8	4.4	< 0.05
Explained variance (%)	21.3	0.8	37.3	1.5	39.1

From the overall influence of the individual factors on the total variance, it can be seen that there is a large disproportion between the influences of the individual factors. In particular, there is an enormous contribution from factors I, III, V and a negligible contribution from the other factors. This disproportion is due to the PMF model itself, which is basically a weight model, and given the high content of Fe and Mn in relation to other elements in the soil, it is the basis for the cause of this disproportion in influence.

Arsenic has the greatest influence on the first factor with over 59%, while the influence of Fe is also present (21.8%). The second factor is most strongly influenced by Sb (77%), followed by Hg (40.3%) and Pb (38.5%). The third factor is mainly influenced by Mn and almost equally by Cr, Cu, Fe, Ni, Zn and Hg. The fourth factor is most strongly influenced by Co, Pb, Cd and Zn which account for 57%, 49.8%, 44.9% and 41.9% respectively, while the influence of As (24.8%) is lower. The fifth factor is dominated by Cr and Ni with 47.5% and 37.4% respectively (Table 2).

**Fig. 5:**
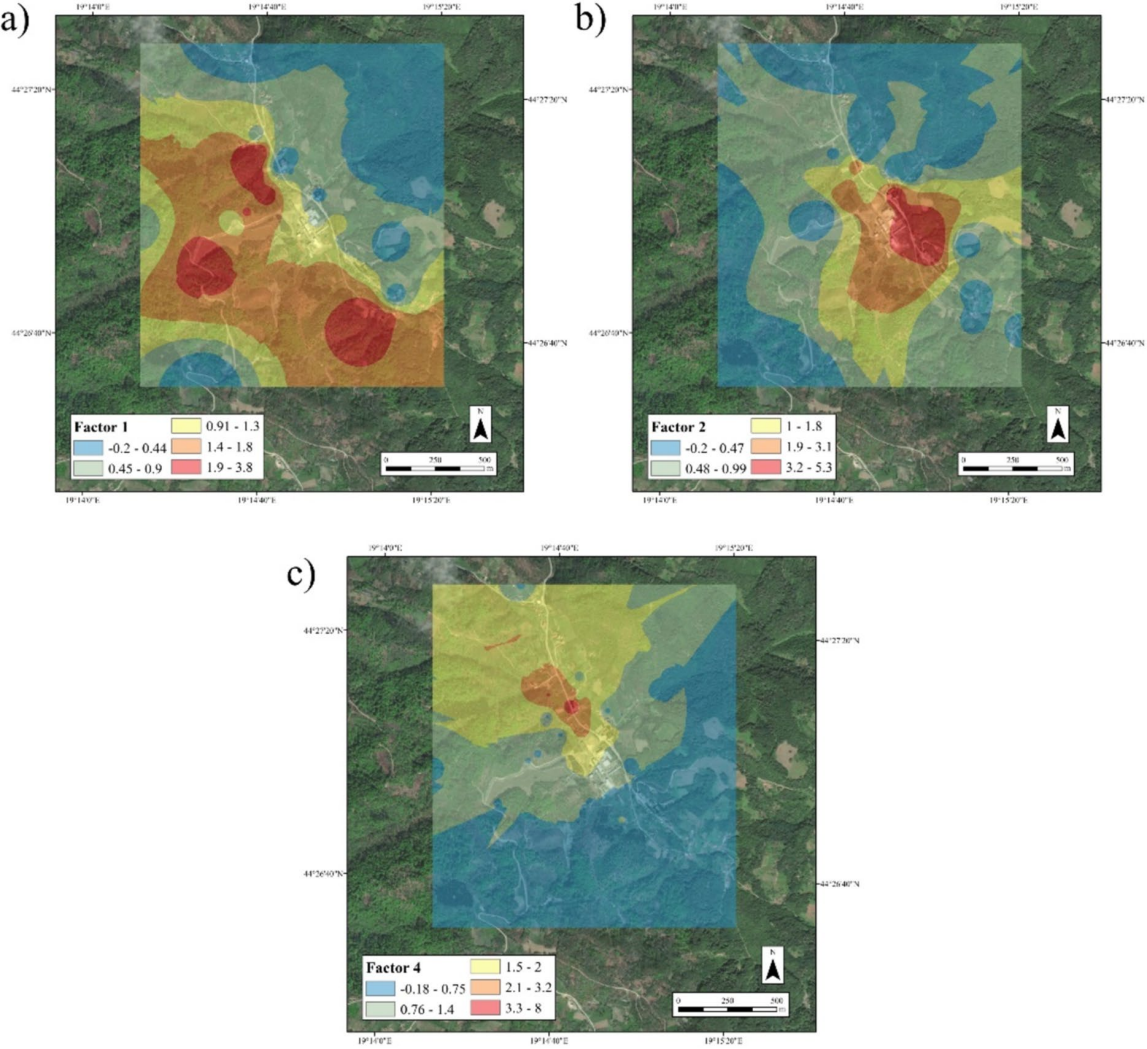
Spatial influence for PMF factors. **a** I factor **b** II factor **c** IV factor

The influence of Factor I is quite widespread, with three points of influence to be observed. The first is located in the south-eastern part of the study area, upstream along the Štira River, well before the recycling factory and the tailings ponds itself, the second near the antimony mine and the third downstream of the tailings ponds. The influence of factor II was found directly at the battery factory. The influence of factor IV was determined downstream, northwest of the tailings pond (Fig. 5a, b, c). The influence of factor III was determined in a wide area immediately downstream of the factory in the south-eastern part of the study area, while the influence of factor V is mainly distributed in a wider area on the right bank of the Štira River (Fig. S4). It is clear that the latter two factors are determined by the geological substrate, given the strong influence of Fe, Cr and Ni.

### Health risk

**Fig. 6:**
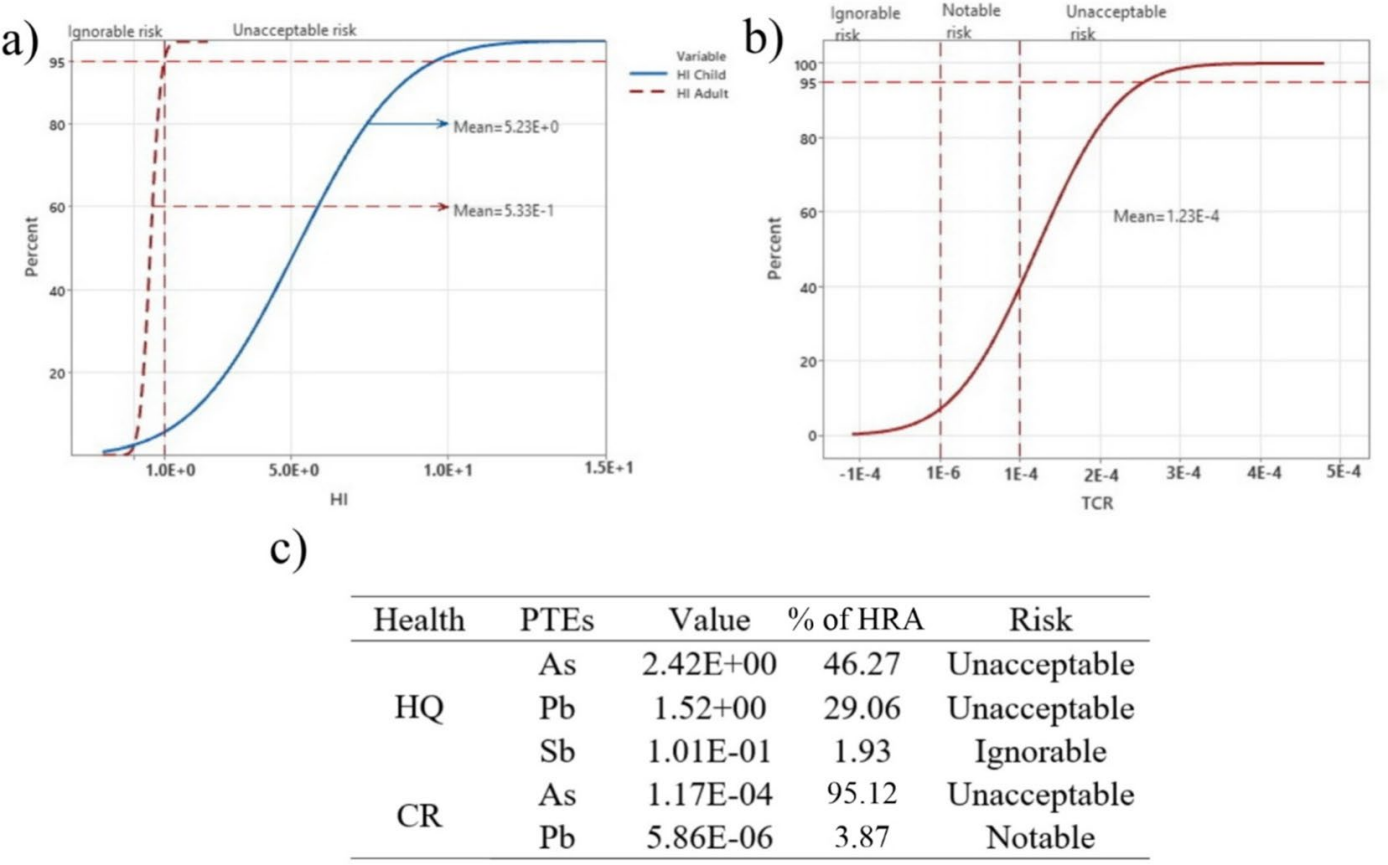
Probability distribution for **a** non-carcinogenic health risk (HI) for children and adults, **b** total carcinogenic risk (TCR) and **c** hazard quotients HQ for As, Pb and Sb for children, and carcinogenic risk (CR) for As and Pb (at 95% confidence level; UNA-unacceptable risk; NOT-notable risk; IGN-ignorable risk)

Based on the percentage probability for the child population, the mean value of HI is 5.23E + 0, while the highest value at 95% is 9.45E + 0. It was also found that 5.61% of the probability is below 1E + 0, which means that there is a 94.39% probability of a non-cancer risk in the area in question. Furthermore, the mean value for adults is 5.33E-1 and all values are below the value 1E + 0 with a probability of 95%, which clearly indicates the low influence of these elements on the HQ of adults (Fig. 6a).

#### Hazard index for individual PTEs

The mean HQ values at the 95% probability level for As, Pb and Sb for children are 2.42E + 0, 1.52E + 0 and 1.01E-1 respectively. For children, the total health risk (HRA) for the three studied elements 77.26% for HQ and 46.27% for As alone, indicating the importance of these three elements as the main factors affecting children’s health. (Fig. 6c). For adults, all values are below 1 and were determined for As, Pb and Sb: 2.58E-1, 1.44E-1 and 9.64E-2 respectively.

A probability of 39.27% was determined for TCR values below 1E-4, while only 7.10% were below the value of 1E-6 (Fig. 6b). A mean value of 1.23E-4 was determined (Fig. 6b), while the mean value for As was 1.17E-4 and for Pb 5.86E-6. In the case of TCR, it can be clearly seen that CR is 95.12% for As and 98.99% for Pb. (Fig. 6c).

#### Source oriendeted health risk assessment

**Fig. 7:**
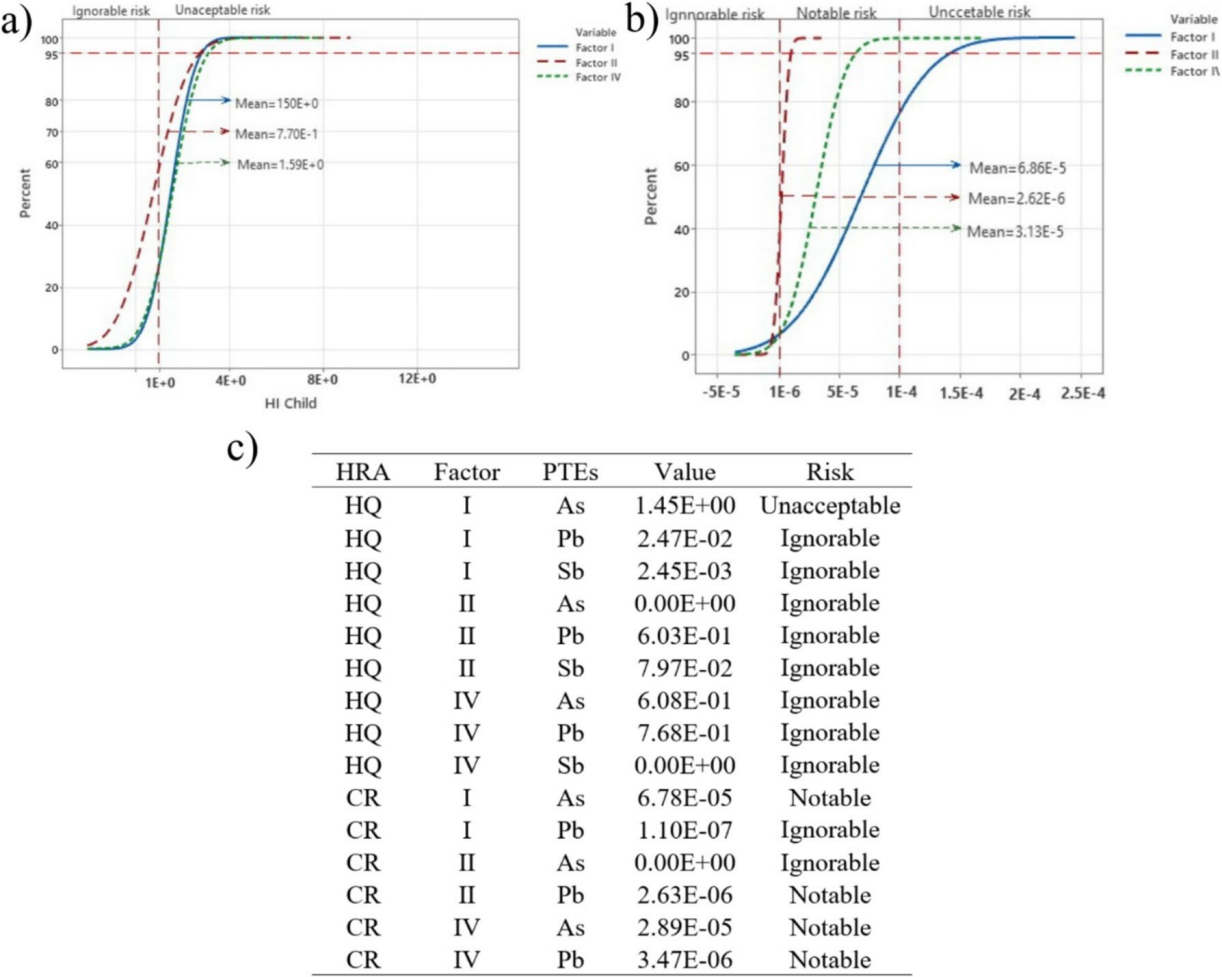
**a** The blue, red and green curves represent the probability distribution of Factor 1, Factor 2 and Factor 4 of the hazard index (HI) for each source, **b** of the total carcinogenic risk (TCR) for each source, **c** carcinogenic risk (CR) for As and Pb and the hazard quotients (HQ) for As, Pb and Sb for children (at 95% confidence level; UNA-unacceptable risk; NOT-notable risk; IGN-ignorable risk)

Taking into account the fact that As and Pb, and to some extent also Sb, are anthropogenic pollutants, only three factors were considered: I, II and IV. The highest values for the hazard index at 95% confidence interval were found for children: 2.80E + 0, 2.72E + 0 and 3.06E + 0, while the mean values for factors I, II and IV were 1.50E + 0, 7.70E- 1, i.e. 1.59E + 0. It was also found that 27.13% of the probability for factors I and IV are without risk, while for factor II the probability is 57.30% without risk (Fig. 7a). For adults, the values for the same factors at 95% probability are 3.30E-1, 2.6E-1 and 3.30E-1, while the mean values for factors I, II and IV are 1.72E-1, 7.35E-2 and 1.65E-1, respectively.

The highest values for TCR at 95% probability for factors I, II and IV are 1.40E-4, 1.05E-5 and 6.35E-5, while the mean values are 6.86E-5, 2.62E-6 and 3.13E-5. It is noticeable that for factor I only 7.72% of the results obtained belong to the risk-free values, while 68.3% belong to the group with significant risk. For risk-free factors II and IV, it was found that only 6.72% and 7.13% respectively belong to this group (Fig. 7b).

Looking at the values of the analysed elements by factor, the non-cancer risk for As at factor I is unacceptable. For Pb, the value for factor IV is slightly higher than for factor II. With regard to the cancer risk, it can be seen that As has slightly higher values for factors I and IV than Pb, although both values fall into the group of appreciable risks. For Pb, it was also found that the values for factor IV are slightly higher than for factor II (Fig. 7c).

## Discussion

The focus of the research is on the investigation of Pb, As and Sb, as their presence in the children’s blood was detected. Lead was selected because it was found that the mean blood lead level (BLL) in children is 17.5 µg dl^−1^ (Mandić-Rajčević et al., 2018), with BLL at 5 µg dl^−1^ being the threshold value (Centres for Disease Control, 2022; Council, 1993). Antimony was selected because ore deposits of this element are found here (MEP, 2016), while As is a very strong pollutant and a clear companion of antimony ore deposits characteristic of this area (Belanović Simić et al., 2023; Cakmak et al., 2014). Their inclusion is also justified by the relatively high concentration of these elements with a high percentage of limit value exceedances. All maximum values and for As also the mean value were above the intervention values (Tables 1, S4). Apart from these high values, the first indication of anthropogenic influence on these elements can be found in the high coefficients of variation (Karim et al., 2014), with the highest (130%) found for Sb and the lowest (68.33%) for As (Table 1). This percentage ratio also indicates a relative uniformity of As content throughout the area compared to other elements, also recognising that a significantly higher concentration of Pb and Sb was found between the battery processing factories, while quite high spatial uniformity was found for As, especially near the watercourses of the Štira River (Fig. S1, Table S4).

In contrast to the classical methods of perception, network analysis (NA), with its net-like interweaving of elements, mimics natural processes more closely and simplifies their summarization and definition, so that two basic groups were formed on its basis. The first shows the influence of the soil on certain elements, where the associated elements are Fe as a reference soil element and Ni, which is characteristic of the soil in this area (Čakmak et al., 2023; MEP, 2016). The second group consists of elements that are strongly influenced by the anthropogenic-atmospheric factor, as it contains all three analysed elements, As, Pb and Sb. In this group, Pb plays the role of the element with the greatest influence, that is, Pb is the element with the most established bonds. Apart from the obvious associations of Pb with atmospheric elements, its association with Cu indicates its origin from the soil, as does Cu as a companion of Sb ore deposits (Bech et al., 2012). In addition to the association of Sb with Cu, an association with Hg was also noted, as Hg is a companion of the same ore deposits (Belanović Simić et al., 2023), and another association of Hg with Mn links it to the soil, apart from the noted atmospheric anthropogenic origin. Interestingly, although As nominally belongs to the group of anthropogenic-atmospheric elements, it is not closely related to any element except Cd, which argues for its possible origin from a wider area than the studied one, which is also supported by the negative relationship with the typical soil element, i.e. Cr (Fig. 2a, b).

CoDA clearly shows the strongest influence of Sb in the geological base of the studied area, where its location is strictly determined by the mine (Fig. 1), where the association with Hg, Pb and partially As is established, which is common for antimony mines (Bech et al., 2012; Belanović Simić et al., 2023) in the geological substrate (Fig. 3a, b). However, the effect of Sb is also pronounced in the vicinity of the battery factory itself, where the highest levels of this element were measured in the soil, which is a consequence of anthropogenic pollution (Fig. 1, Table S4).

The PCA clearly shows 4 factors, two of which are of geological origin due to their spatial distribution and the significant influence of Fe as a reference element (Čakmak et al., 2023) (Fig. S3a, b; Table S5). The other two factors, which are under anthropogenic influence, are significantly influenced by Pb, clearly indicating a dual source of its origin (Table S5). Factor I has a significant influence from two of the three elements analysed, namely As and Pb, while other elements are either companions of Pb, such as Zn (Zhou et al., 2019), or companions of Sb mines, such as Cu and Cd (Bech et al., 2012). The conspicuous absence of Sb in this factor is a consequence of the perfected technological process for extracting Sb from the ore (IKS, 2012), which at the same time meant that Sb was not transported from the tailings ponds by the wind to any great extent, i.e. it clearly shows the influence of the tailings pond, i.e. the contaminated site (Fig. 4a). Factor IV with a clear influence of Sb, Pb and Hg indicates the history of the smelter itself, where Hg is a remnant of ore smelting as Hg evaporates in its composition (Fu et al., 2010). The pollution that has existed since the 1990s due to the conversion of the ore smelter into a battery smelter (IKS, 2012) is evident by the increased influence of Sb contained in the batteries (John Ogheneortega and Karrem, 2012), which is a clear indication of the current pollution in the vicinity of the smelter (Fig. 4b).

With regard to the PMF model, it should be emphasised that the proportion of the individual factors in the total sum of variability is not valid for the soil, as it is based on weight percentages, so that Fe-containing factors have a significantly larger proportion, which can be a disadvantage of this method (Table 2). In contrast to PCA, five factors were selected for the PMF model. The reason for this is the higher accuracy of determining point sources in fluid systems (Čakmak et al., 2023; Zhi et al., 2016). As was clearly seen in Factor I (Table 2), the distribution of this factor is very broad (Table 1, Fig. 5a). The even distribution of this factor and of As itself is due to other Sb mines in the vicinity, e.g. Stolice, which is 8.35 km from this area, as well as the Štira mine, which is located 1 km upstream along the Štira River. This indicates a very wide distribution of Sb deposits, which is also confirmed by the results obtained with CoDA (Fig. 3a, b). Considering the easy transport of As through rivers, due to its reduced soluble form, and through the atmosphere (Čakmak et al., 2018; Pejović et al., 2017), its large spatial impact becomes evident. Similar to the PCA model, two independent sources were identified for Pb that spatially coincide with those separated in PCA, namely Factor II, which indicates the factor of smelting with a strong influence of Sb, Hg and Pb in the immediate vicinity of the smelter, and Factor IV with the dominant influence of Pb, where the effects of Co, Cd and Zn were also observed (Table 2; Fig. 5b, c). The other two factors are the geological base, where the influence of Fe was observed in addition to Cr, and for Factor V, Ni and Cr were the dominant influences of the PTEs characteristic of the geological base of this area (Table 2; Fig. S4a, b).

The data on the detection of elevated Pb levels in the blood of children in the area of the Zajače settlement already points to the problem of pollution. This pollution can be defined as atmospheric, which is very likely due to the operation of the battery smelter (Mandić-Rajčević et al., 2018), but it was also necessary to investigate the impact of soil on the possible occurrence of chronic diseases, as well as the likelihood of cancer occurrence, especially having a long-term effect of the tested elements in the soil (Pavlović et al., 2021; Sieghardt et al., 2005). Based on the real data from 30 soil samples, it is noticeable that a very large percentage of the samples for As (76.66%) and Pb (43.33%) are above the permissible value for HI, while one sample (no. 19) had the second highest value for As (5.94E + 00) and the highest value for Pb (7.28E + 00). It is important to emphasise that the influence of factors II and IV on Pb was the highest at this point with values of 2.80E + 00 and 3.63E + 00, respectively, attesting to the strong influence of both sources on chronically Pb-exposed children. For As, it is characteristic that the highest HI value was determined in sample no. 3 (7.69E + 00) (Table S6). Significant for the cancer risk is that for As it was determined that 53.33% of the samples fall into the “unacceptable risk” category, while all the others have a notable risk, while for Pb it was found that 66.66% of the samples fall into the “notable risk” category, while for the rest no risk was found, which also indicates an area extremely polluted with these elements.

The use of MSC allowed us to determine a percentage for the probability of health risk. The mean HI value for children is about five times higher than acceptable probability at 95%, with As accounting for 46.27%, Pb 29.06% and Sb, whose mean value does not exceed the unacceptable risk, only 1.93%. This relationship clearly shows that As and Pb from soil are the most important potential causes of chronic diseases in children (Fig. 6a, c). For As, it is characteristic that the mean value of Factor I is also above the acceptable risk level. It is also significant that factor IV with the proportion of As and Pb has the highest mean value at the 95% probability level (1.59E + 00) (Fig. 7a). In adults, as with HI, no increase in the mean value above the safe risk level was observed, which is logical, given the resistance of older organisms to the PTE effect and the time period of the potential effect (Fig. S5).

Regarding the possible occurrence of cancer, it is clear that the mean value of the TCR is at an unacceptable level (1.23E-4) (Fig. 6b), which was also found for As (1.17E-4) due to its high carcinogenic effect (Čakmak et al., 2020). Lead poses a significant risk (Fig. 6, 7). A notable risk was also found for all three observed components under anthropogenic influence (I, II and IV), indicating a serious threat to the area (Fig. 7c). Small differences in the mean values of factors II (2.63E-6) and IV (3.47E-6) with respect to Pb indicate that the impact of historical and current pollution on the population of the area is almost equal. The current pollution is much more dangerous for the health of the population compared to the historical pollution (Fig. 7c).

## Conclusions

The results of the soil analysis in the area of the Zajača mine and smelter show that the determined levels of As, Pb and Sb exceed the target value for the studied area, with the highest ecological risk identified for Pb and the lowest for Sb. Based on a network analysis, it was determined that these elements originate from the atmosphere and that they are caused by anthropogenic influences in the region, which is strongly influenced by Sb ore and its compounds. The receptor methods showed that As originates from a larger area, while the sources of Pb in the soil originate from two identified sources. The levels of these two elements in the soil indicate that the As content is unacceptably high in terms of carcinogenic diseases, while the Pb content is significantly high.

This shows that the previously identified air pollution is not the only factor affecting the health of the population of this settlement, but that current and historical soil pollution also have a major impact. Given the nature of the pollution, this will continue over a long period of time. It is clear that any form of exploitation of ore deposits carries some pollution risk, but the exploitation of Sb deposits complicates this option many times over, considering that the raw materials of Sb compounds are closely related to other PTEs and their combined effect on human health can be associated with serious consequences. Therefore, in the exploitation of such mines and especially in the further processing of this material, as well as in the eventual processing of waste, great attention must be paid to the ecological techniques of exploitation, and it is necessary to establish constant monitoring of the subsoil and constant monitoring of the health of people exposed to these impacts.

## Supplementary Information

Below is the link to the electronic supplementary material.

Supplementary file1 (DOCX 1986 KB)

## Data Availability

No datasets were generated or analysed during the current study.

## Declarations

### Confict of interest

The authors declare that they have no confict of interest.
