# Supplementary material for: Development of optimisation methods to identify sources of pollution and assess potential health risks in the vicinity of antimony mines
Source: Environ Geochem Health. 2025 Feb 11;47(3):73. doi: 10.1007/s10653-025-02369-0 (PMC11814042; doi:10.1007/s10653-025-02369-0)
Supplement: Supplementary file 1 — Supplementary file1 (DOCX 1986 KB) [file 10653_2025_2369_MOESM1_ESM.docx]

**Supplementary Information**

For

**Development of optimisation methods to identify sources of pollution and assess potential health risks in the vicinity of antimony mines**

Dragan Čakmak^a*^, Veljko Perović^a^, Dragana Pavlović^a^, Marija Matić^a^, Darko Jakšić^b^, Samat Tanirbergenov^c^, Pavle Pavlović^a^

^a^Department of Ecology, Institute for Biological Research 'Siniša Stanković', University of Belgrade, Bulevar despota Stefana 142, 11108 Belgrade, Serbia

^b^Institute of Agricultural Economics, Volgina 15, 11060 Belgrade, Serbia

^c^Department of Agrochemistry and Soil Ecology, U.Uspanov Kazakh Research Institute of Soil Science and Agrochemistry, 75 Al-Farabi Ave, 050060 Almaty, Kazakhstan.

E-mail: [dragan.cakmak@ibiss.bg.ac.rs](mailto:dragan.cakmak@ibiss.bg.ac.rs)

Tel: +38111 2078371

Fax: +381 11 2761433

*Corresponding author, <https://orcid.org/0000-0002-2888-494X>

**Tables:**

Table S1. Value for target value (SW), intervention value (IW) and constants (A, B, C) for As, Pb and Sb

Table S2. Description and values of all parameters associated with health risk assessment for PTEs in soils

Table S3. Relative bioavailability factor (RBA), oral reference dose (RfDo), dermal absorption fraction (ABSd), gastrointestinal absorption (GIABS), inhalation reference concentration (RfC), oral slope factor (CSFo) and inhalation unit risk (IUR) values

Table S4. Content of potentially toxic elements (PTEs) in individual samples

Table S5. Rotated Component Matrix^a^

Table S6. Non-carcinogenic health risk (HI) for children

Table S7. Total carcinogenic risk (TCR)

**Figures:**

Figure S1. Values above target and intervention values for a) As b) Sb c) Pb

Figure S2. Ecological risk for a) As, b) Sb, c) Pb

Figure S3. Spatial influence a) II factor b) III factor

Figure S4. Spatial influence a) III factor b) V factor

Figure S5. Probability distribution for non-carcinogenic health risk (HI) for adults

**Table S1.** Value for target value (SW), intervention value (IW) and constants (A, B, C) for As, Pb and Sb

|  | SW | IW | A | B | C |
| --- | --- | --- | --- | --- | --- |
| mg kg^-1^ | | | / | | |
| As | 23.90* | 55.00 | 15.00 | 0.40 | 0.40 |
| Pb | 47.78* | 530.00 | 50.00 | 1.00 | 1.00 |
| Sb | 1.04* | 15.00 | / | / | / |

*Background value (MEP 2016)

**Table S2.** Description and values of all parameters associated with health risk assessment for PTEs in soils

| Symbol | Parameters (units) | | Values | | References |
| --- | --- | --- | --- | --- | --- |
| C | | PTE concentration (mg/kg) | | Site specific | |
| IRSres-c | Resident Soil Ingestion Rate - Child (mg day^-1^) | | 200 | | USEPA, 2020 |
| IRSres-a | Resident Soil Ingestion Rate - Adult (mg day^-1^) | | 100 | | USEPA, 2020 |
| RBA | relative bioavailability factor | | Arsenic=0.6 All Others=1 | | USEPA, 2020 |
| EFres | Resident Exposure Frequency Adult, Child (days year^-1^) | | 350 | | USEPA, 2020 |
| EDres-c | Resident Exposure Duration - child (years) | | 6 | | USEPA, 2020 |
| EDres-a | Resident Exposure Duration - adult (years) | | 20 | | EDres (26 years)-EDres-c (6 years) USEPA, 2020 |
| BWres-c | Resident Body Weight - child (kg) | | 15 | | USEPA, 2020 |
| BWres-a | Resident Body Weight - adult (kg) | | 80 | | USEPA, 2020 |
| ATres-c | Averaging time - resident child (days) | | 365 x EDres-c=2190 | | USEPA, 2020 |
| ATres-a | Averaging time - resident adult (days) | | 365 x EDres =7300 | | USEPA, 2020 |
| RfDo | Chronic Oral Reference Dose (mg kg^-1^-day) | | Contaminant specific | | Table S3 |
| SAres-c | Resident surface area soil - child (cm^2^ day^-1^) | | 2373 | | USEPA, 2020 |
| SAres-a | Resident surface area soil - adult (cm^2^ day^-1^) | | 6032 | | USEPA, 2020 |
| AFa | Skin adherence factor - adult (mg cm^-2^) | | 0,07 | | USEPA, 2020 |
| AFc | Skin adherence factor - child (mg cm^-2^) | | 0,2 | | USEPA, 2020 |
| ABSd | Fraction of contaminant absorbed dermally from soil (unitless) | | Contaminant specific | | Table S3 |
| GIABS | Fraction of contaminant absorbed in gastrointestinal tract (unitless) Note: if GIABS is >50% then it is set to 100% for calculation of dermal toxicity values | | Contaminant specific Inorganic default=1.0 VOC default=1.0 SVOC default=1.0 | | Table S3 |
| RfC | Chronic Inhalation Reference Concentration (mg m^-3^) | | Contaminant specific | | Table S3 |
| PEF | Particulate Emission Factor - Minneapolis (m^3^ kg^-1^) | | 1.36 x 109(region-specific) | | USEPA, 2020 |
| IFSres-adj | Resident Soil Ingestion Rate - age-adjusted (mg kg^-1^) | | Calculated using the age adjusted intake factors equation 36750 | | USEPA, 2020 |
| DFSres-adj | Resident soil dermal contact factor- age-adjusted (mg kg-^1^) | | Calculated using the age adjusted intake factors equation 103390 | | USEPA, 2020 |
| CSFo | | Oral Slope Factor (mg kg^-1^-day)^-1^ | | Table S3 | |
| IUR | Inhalation Unit Risk (μg m^-3^)^-1^ | | Contaminant specific | | Table S3 |
| LT | Life time (years) | | 76 | | Site specific |
| AT | Averaging time (days) | | 365*LT=27740 (Carcinogenic) | | Site specific |

**Table S3.** Relative bioavailability factor (RBA), oral reference dose (RfDo), dermal absorption fraction (ABSd), gastrointestinal absorption (GIABS), inhalation reference concentration (RfC), oral slope factor (CSFo) and inhalation unit risk (IUR) values

| PTE | RBA | RfDo | ABSd | GIABS | RfC | CSFo | IUR |
| --- | --- | --- | --- | --- | --- | --- | --- |
| As | 0.6 | 0.0003 | 0.03 | 1 | 0.000015 | 1.5 | 0.0043 |
| Cd | 1 | 0.003 | 0.001 | 0.25 | 0.0.00005 | / | 0.0018 |
| Co | 1 | 0.0003 | 0.001 | 1 | 0.000006 | / | 0.009 |
| Cr (III) | 1 | 1.5 | 0.001 | 1 | 0.000006 | / | / |
| Cu | 1 | 0.04 | 0.001 | 1 | 0.0024^b,c^ | / | / |
| Fe | 1 | 0.7 | 0.001 | 1 | / | / | / |
| Mn | 1 | 0.024 | 0.001 | 0.04 | 0.00005 | / | / |
| Ni | 1 | 0.02 | 0.001 | 0.04 | 0.000006 | / | 0.00026 |
| Pb | 1 | 0.0014^a^ | 0.001 | 0.15 | 0.0015^b,c^ | 0.0085^b,c^ | 0.000012^b,c^ |
| Sb | 1 | 0.0004 | 0.001 | 1 | 0.0003 | / | / |
| Zn | 1 | 0.3 | 0.001 | 1 | 0.035^b,c^ | / | / |
| Hg | 1 | 0.0003 | 0.001 | 0.007 | 0.3 | / | / |
| Source | | | | USEPA (2020) | | | |

^a^Jia et al., (2018); ^b^USDOE, (2011); ^c^Čakmak et al., (2020)

**Table S4.** Content of potentially toxic elements (PTEs) in individual samples

| Point | Geographic | | Potentially toxic elements | | | | | | | | | | | |
| --- | --- | --- | --- | --- | --- | --- | --- | --- | --- | --- | --- | --- | --- | --- |
| No. | Lati. | Long. | As | Cd | Co | Cr | Cu | Fe | Mn | Ni | Pb | Sb | Zn | Hg |
|  | x | y | mgkg^-1^ | | | | | | | | | | | |
| 1 | 360148 | 4924223 | 30.79 | 1.15 | 13.90 | 27.91 | 24.58 | 26026.45 | 716.96 | 29.35 | 184.50 | 1.76 | 106.52 | 0.15 |
| 2 | 360285 | 4923757 | 73.53 | 2.08 | 8.05 | 11.77 | 32.87 | 14883.08 | 711.96 | 20.50 | 391.12 | 1.94 | 254.01 | 0.18 |
| 3 | 360287 | 4923666 | 268.48 | 4.84 | 9.55 | 13.27 | 18.49 | 18556.45 | 746.46 | 12.10 | 680.80 | 0.94 | 279.87 | 0.38 |
| 4 | 360283 | 4923554 | 105.18 | 0.65 | 6.90 | 8.61 | 11.33 | 16721.45 | 697.46 | 6.87 | 271.70 | 2.54 | 64.22 | 0.22 |
| 5 | 360450 | 4923490 | 123.93 | 1.82 | 8.67 | 13.82 | 50.43 | 17411.45 | 610.46 | 17.22 | 995.80 | 17.47 | 172.67 | 0.21 |
| 6 | 360348 | 4923463 | 108.38 | 0.92 | 8.20 | 12.22 | 13.36 | 15211.45 | 635.46 | 8.98 | 504.80 | 6.09 | 64.87 | 0.43 |
| 7 | 360235 | 4923369 | 115.18 | 0.46 | 3.90 | 15.56 | 12.88 | 18071.45 | 102.06 | 9.63 | 135.60 | 0.80 | 30.94 | 0.14 |
| 8 | 360184 | 4923331 | 65.88 | 0.60 | 5.14 | 12.81 | 11.25 | 14411.45 | 219.21 | 8.02 | 585.30 | 1.09 | 42.73 | 0.06 |
| 9 | 359702 | 4923292 | 44.16 | 0.54 | 6.54 | 21.02 | 11.21 | 19858.08 | 327.71 | 12.85 | 66.67 | 0.39 | 60.46 | 0.09 |
| 10 | 360021 | 4922488 | 11.95 | 0.67 | 12.57 | 23.92 | 18.07 | 24543.08 | 600.46 | 22.52 | 43.05 | 0.31 | 71.66 | 0.10 |
| 11 | 360032 | 4923120 | 166.48 | 0.59 | 8.36 | 14.01 | 17.12 | 18481.45 | 466.51 | 10.47 | 500.80 | 1.93 | 85.22 | 0.12 |
| 12 | 360660 | 4923032 | 108.18 | 1.79 | 12.85 | 22.66 | 18.76 | 21178.08 | 888.96 | 19.94 | 606.92 | 4.88 | 92.46 | 0.39 |
| 13 | 360828 | 4922821 | 153.73 | 0.57 | 11.03 | 10.37 | 15.16 | 20391.45 | 517.46 | 13.50 | 119.40 | 1.73 | 74.62 | 0.28 |
| 14 | 360883 | 4922851 | 102.18 | 0.59 | 11.26 | 12.27 | 17.69 | 19886.45 | 896.46 | 16.36 | 88.70 | 1.79 | 78.92 | 0.25 |
| 15 | 360964 | 4922833 | 116.03 | 1.13 | 14.82 | 20.53 | 28.42 | 29208.08 | 605.96 | 25.57 | 158.02 | 1.55 | 126.01 | 0.25 |
| 16 | 360288 | 4924157 | 50.23 | 2.04 | 11.55 | 19.37 | 25.77 | 12148.08 | 533.46 | 26.53 | 449.92 | 0.59 | 95.36 | 0.27 |
| 17 | 360442 | 4923757 | 64.18 | 0.79 | 5.53 | 13.76 | 12.34 | 13543.08 | 277.71 | 12.31 | 138.12 | 0.00 | 53.51 | 0.06 |
| 18 | 360440 | 4923654 | 27.08 | 0.80 | 6.70 | 10.27 | 11.88 | 14716.45 | 269.81 | 11.32 | 270.05 | 0.00 | 72.67 | 0.01 |
| 19 | 360408 | 4323606 | 207.48 | 4.35 | 9.89 | 28.38 | 49.26 | 19801.45 | 492.91 | 48.68 | 1987.80 | 6.36 | 613.97 | 0.25 |
| 20 | 360463 | 4923575 | 66.98 | 1.72 | 11.61 | 22.20 | 30.15 | 23263.08 | 1080.96 | 23.15 | 597.92 | 2.88 | 243.51 | 0.26 |
| 21 | 360587 | 4923587 | 57.73 | 0.85 | 13.81 | 27.30 | 23.83 | 25688.08 | 1089.46 | 25.81 | 116.57 | 1.09 | 88.06 | 0.17 |
| 22 | 360658 | 4923566 | 50.43 | 0.91 | 12.58 | 20.72 | 20.11 | 21776.45 | 834.96 | 20.81 | 164.85 | 0.49 | 90.32 | 0.13 |
| 23 | 360840 | 4923693 | 24.09 | 0.70 | 14.05 | 35.11 | 19.78 | 26306.45 | 517.96 | 34.26 | 66.60 | 1.37 | 92.02 | 0.06 |
| 24 | 360922 | 4923757 | 32.97 | 1.26 | 11.09 | 30.42 | 17.65 | 20683.08 | 755.96 | 29.47 | 148.62 | 1.30 | 99.01 | 0.28 |
| 25 | 360602 | 4923461 | 86.88 | 1.67 | 11.56 | 38.39 | 37.39 | 22923.08 | 646.96 | 49.00 | 974.92 | 8.17 | 203.36 | 0.49 |
| 26 | 360700 | 4923313 | 58.08 | 1.90 | 18.49 | 29.20 | 34.17 | 33378.08 | 1658.46 | 39.36 | 893.92 | 6.58 | 107.66 | 0.81 |
| 27 | 360762 | 4923221 | 87.58 | 2.17 | 9.27 | 29.75 | 28.10 | 30273.08 | 255.66 | 30.46 | 832.42 | 13.49 | 85.11 | 0.62 |
| 28 | 360952 | 4923205 | 16.47 | 0.55 | 15.86 | 27.12 | 25.06 | 26876.45 | 930.96 | 29.91 | 68.35 | 1.33 | 94.67 | 0.14 |
| 29 | 360991 | 4922950 | 29.14 | 1.05 | 12.86 | 24.47 | 23.67 | 25258.08 | 600.96 | 28.16 | 169.52 | 1.49 | 118.31 | 0.17 |
| 30 | 361236 | 4927776 | 99.98 | 0.95 | 11.95 | 15.60 | 24.10 | 24416.45 | 627.96 | 25.24 | 117.85 | 1.89 | 140.17 | 0.18 |

**Table S5.** Rotated Component Matrix^a^

|  | Component | | | |
| --- | --- | --- | --- | --- |
|  | 1 | 2 | 3 | 4 |
| As | 0.705 | -0.518 | 0.020 | 0.184 |
| Cd | 0.899 | 0.007 | 0.135 | 0.164 |
| Co | -0.020 | 0.516 | 0.801 | -0.068 |
| Cr | 0.030 | 0.879 | 0.258 | 0.116 |
| Cu | 0.590 | 0.509 | 0.110 | 0.425 |
| Fe | -0.150 | 0.554 | 0.633 | 0.171 |
| Mn | 0.092 | 0.115 | 0.893 | 0.025 |
| Ni | 0.345 | 0.851 | 0.283 | 0.144 |
| Pb | 0.775 | 0.151 | -0.083 | 0.518 |
| Sb | 0.212 | 0.155 | -0.044 | 0.927 |
| Zn | 0.944 | 0.248 | -0.033 | -0.011 |
| Hg | 0.202 | 0.008 | 0.587 | 0.677 |
| Extraction Method: Principal Component Analysis.  Rotation Method: Varimax with Kaiser Normalization. | | | | |
| ^a^Rotation converged in 7 iterations.  KMO Test 0.522 | | | | |

**Table S6.** Non-carcinogenic health risk (HI) for children

| HI | As | Pb | Sb | THI |
| --- | --- | --- | --- | --- |
| 1 | 8.82E-01 | 6.76E-01 | 5.71E-02 | 3.15E+00 |
| 2 | 2.11E+00 | 1.43E+00 | 6.29E-02 | 4.69E+00 |
| 3 | 7.69E+00 | 2.49E+00 | 3.06E-02 | 1.15E+01 |
| 4 | 3.01E+00 | 9.95E-01 | 8.26E-02 | 5.12E+00 |
| 5 | 3.55E+00 | 3.65E+00 | 5.67E-01 | 8.86E+00 |
| 6 | 3.11E+00 | 1.85E+00 | 1.98E-01 | 6.19E+00 |
| 7 | 3.30E+00 | 4.97E-01 | 2.59E-02 | 4.40E+00 |
| 8 | 1.89E+00 | 2.14E+00 | 3.53E-02 | 4.69E+00 |
| 9 | 1.27E+00 | 2.44E-01 | 1.28E-02 | 2.38E+00 |
| 10 | 3.42E-01 | 1.58E-01 | 9.99E-03 | 1.88E+00 |
| 11 | 4.77E+00 | 1.83E+00 | 6.28E-02 | 7.66E+00 |
| 12 | 3.10E+00 | 2.22E+00 | 1.59E-01 | 6.98E+00 |
| 13 | 4.40E+00 | 4.37E-01 | 5.61E-02 | 6.08E+00 |
| 14 | 2.93E+00 | 3.25E-01 | 5.82E-02 | 4.71E+00 |
| 15 | 3.32E+00 | 5.79E-01 | 5.03E-02 | 5.52E+00 |
| 16 | 1.44E+00 | 1.65E+00 | 1.91E-02 | 4.19E+00 |
| 17 | 1.84E+00 | 5.06E-01 | 3.25E-05 | 3.01E+00 |
| 18 | 7.76E-01 | 9.89E-01 | 3.25E-05 | 2.50E+00 |
| 19 | 5.94E+00 | 7.28E+00 | 2.06E-01 | 1.46E+01 |
| 20 | 1.92E+00 | 2.19E+00 | 9.36E-02 | 5.81E+00 |
| 21 | 1.65E+00 | 4.27E-01 | 3.53E-02 | 3.85E+00 |
| 22 | 1.44E+00 | 6.04E-01 | 1.60E-02 | 3.52E+00 |
| 23 | 6.90E-01 | 2.44E-01 | 4.46E-02 | 2.41E+00 |
| 24 | 9.45E-01 | 5.44E-01 | 4.21E-02 | 2.87E+00 |
| 25 | 2.49E+00 | 3.57E+00 | 2.65E-01 | 7.70E+00 |
| 26 | 1.66E+00 | 3.27E+00 | 2.14E-01 | 7.61E+00 |
| 27 | 2.51E+00 | 3.05E+00 | 4.38E-01 | 7.17E+00 |
| 28 | 4.72E-01 | 2.50E-01 | 4.31E-02 | 2.52E+00 |
| 29 | 8.35E-01 | 6.21E-01 | 4.85E-02 | 2.91E+00 |
| 30 | 2.86E+00 | 4.32E-01 | 6.13E-02 | 4.73E+00 |

**Table S7.** Total carcinogenic risk (TCR)

| TCR | As | Pb | Sb | TCR |
| --- | --- | --- | --- | --- |
| 1 | 4.19E-05 | 2.42E-06 | 0.00E+00 | 4.44E-05 |
| 2 | 1.00E-04 | 5.13E-06 | 0.00E+00 | 1.05E-04 |
| 3 | 3.65E-04 | 8.94E-06 | 0.00E+00 | 3.74E-04 |
| 4 | 1.43E-04 | 3.57E-06 | 0.00E+00 | 1.47E-04 |
| 5 | 1.69E-04 | 1.31E-05 | 0.00E+00 | 1.82E-04 |
| 6 | 1.48E-04 | 6.63E-06 | 0.00E+00 | 1.54E-04 |
| 7 | 1.57E-04 | 1.78E-06 | 0.00E+00 | 1.59E-04 |
| 8 | 8.97E-05 | 7.68E-06 | 0.00E+00 | 9.74E-05 |
| 9 | 6.01E-05 | 8.75E-07 | 0.00E+00 | 6.10E-05 |
| 10 | 1.63E-05 | 5.65E-07 | 0.00E+00 | 1.69E-05 |
| 11 | 2.27E-04 | 6.57E-06 | 0.00E+00 | 2.33E-04 |
| 12 | 1.47E-04 | 7.97E-06 | 0.00E+00 | 1.55E-04 |
| 13 | 2.09E-04 | 1.57E-06 | 0.00E+00 | 2.11E-04 |
| 14 | 1.39E-04 | 1.16E-06 | 0.00E+00 | 1.40E-04 |
| 15 | 1.58E-04 | 2.07E-06 | 0.00E+00 | 1.60E-04 |
| 16 | 6.84E-05 | 5.91E-06 | 0.00E+00 | 7.43E-05 |
| 17 | 8.74E-05 | 1.81E-06 | 0.00E+00 | 8.92E-05 |
| 18 | 3.69E-05 | 3.54E-06 | 0.00E+00 | 4.04E-05 |
| 19 | 2.82E-04 | 2.61E-05 | 0.00E+00 | 3.09E-04 |
| 20 | 9.12E-05 | 7.85E-06 | 0.00E+00 | 9.90E-05 |
| 21 | 7.86E-05 | 1.53E-06 | 0.00E+00 | 8.01E-05 |
| 22 | 6.86E-05 | 2.16E-06 | 0.00E+00 | 7.08E-05 |
| 23 | 3.28E-05 | 8.74E-07 | 0.00E+00 | 3.37E-05 |
| 24 | 4.49E-05 | 1.95E-06 | 0.00E+00 | 4.69E-05 |
| 25 | 1.18E-04 | 1.28E-05 | 0.00E+00 | 1.31E-04 |
| 26 | 7.91E-05 | 1.17E-05 | 0.00E+00 | 9.08E-05 |
| 27 | 1.19E-04 | 1.09E-05 | 0.00E+00 | 1.30E-04 |
| 28 | 2.24E-05 | 8.97E-07 | 0.00E+00 | 2.33E-05 |
| 29 | 3.97E-05 | 2.23E-06 | 0.00E+00 | 4.19E-05 |
| 30 | 1.36E-04 | 1.55E-06 | 0.00E+00 | 1.38E-04 |


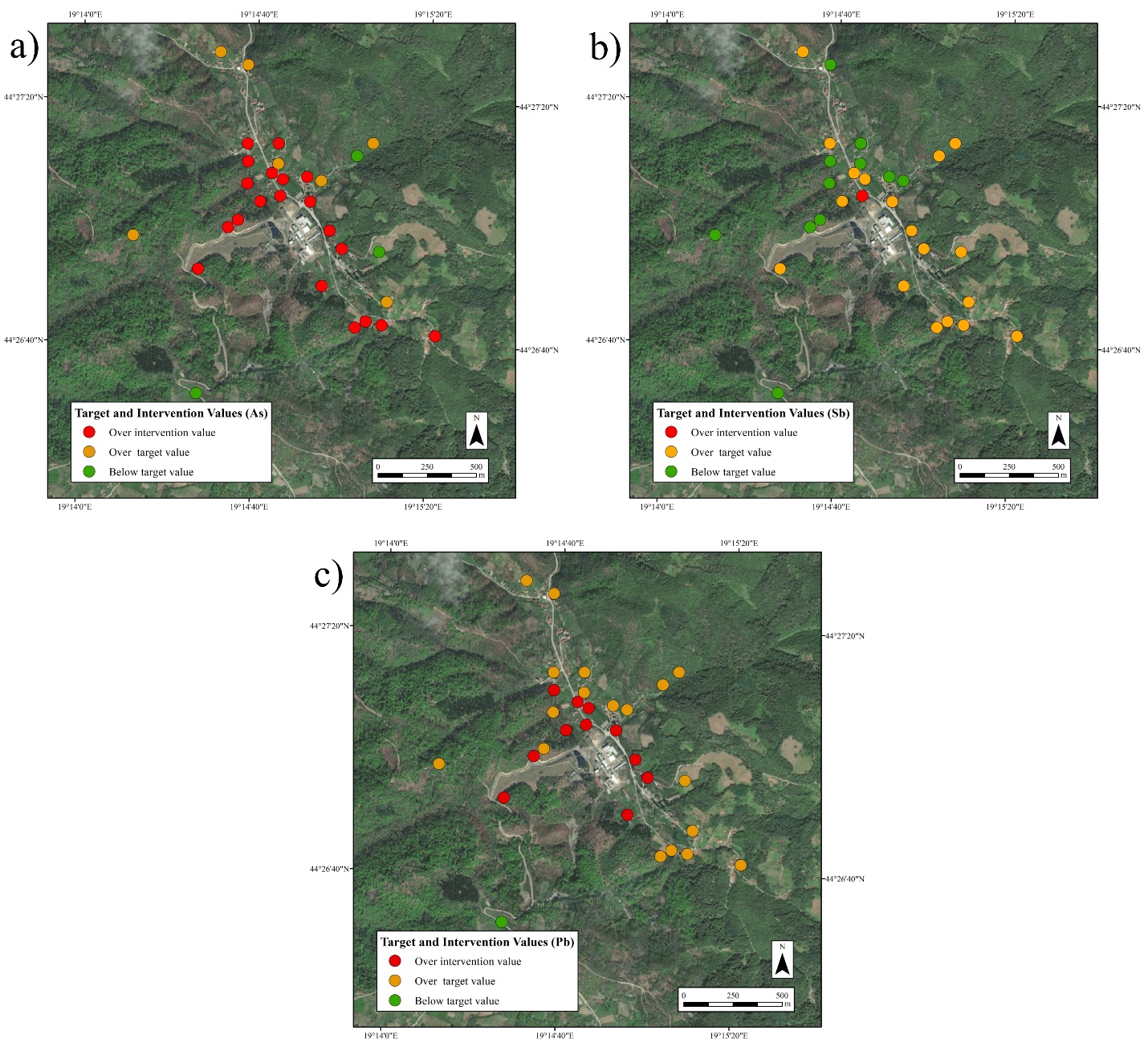
 **Figure S1.** Values above target and intervention values for a) As b) Sb c) Pb


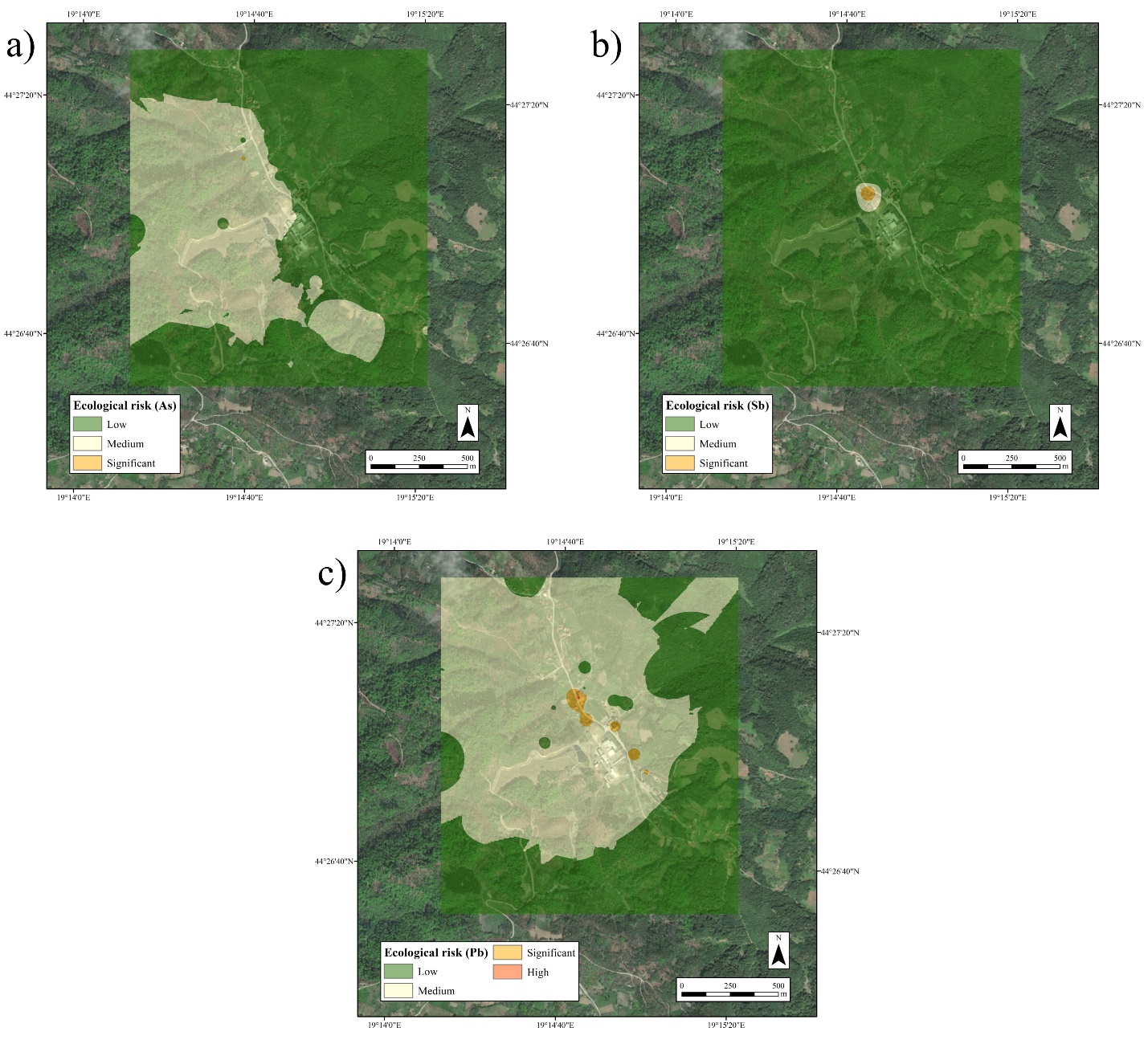


**Figure S2.** Ecological risk for a) As, b) Sb, c) Pb


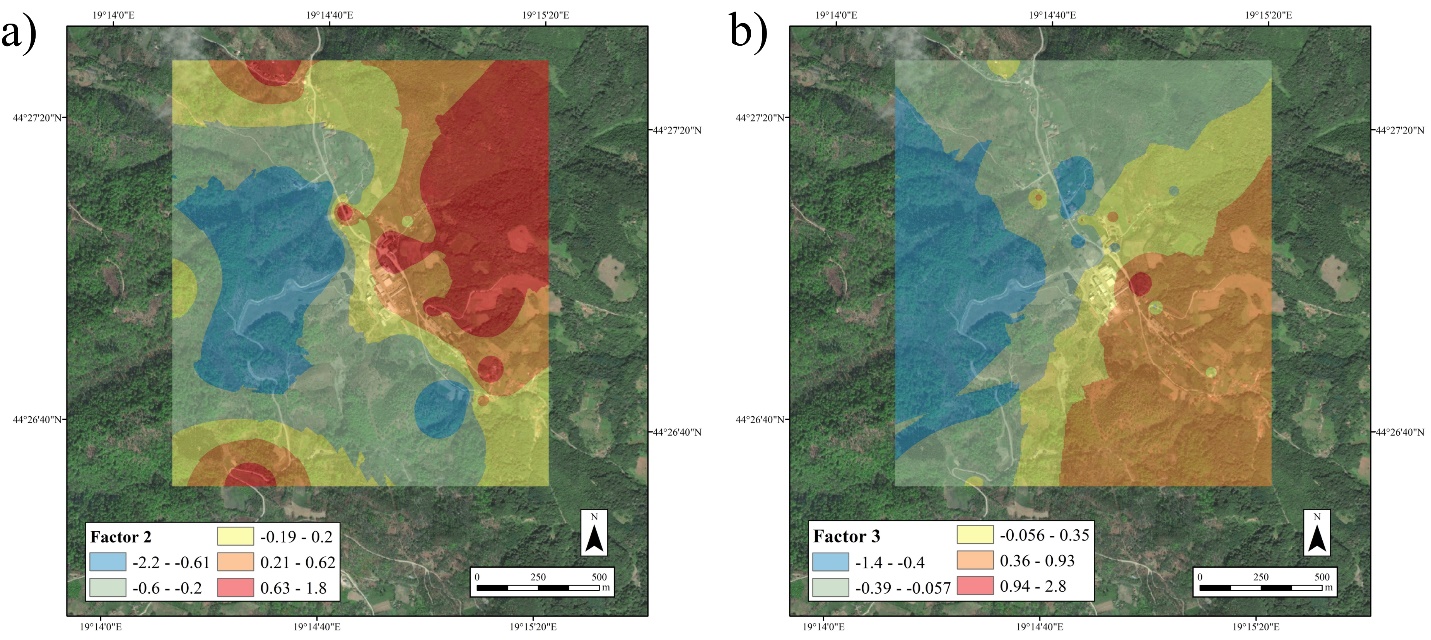


**Figure S3**. Spatial influence for PCA factors a) II factor b) III factor


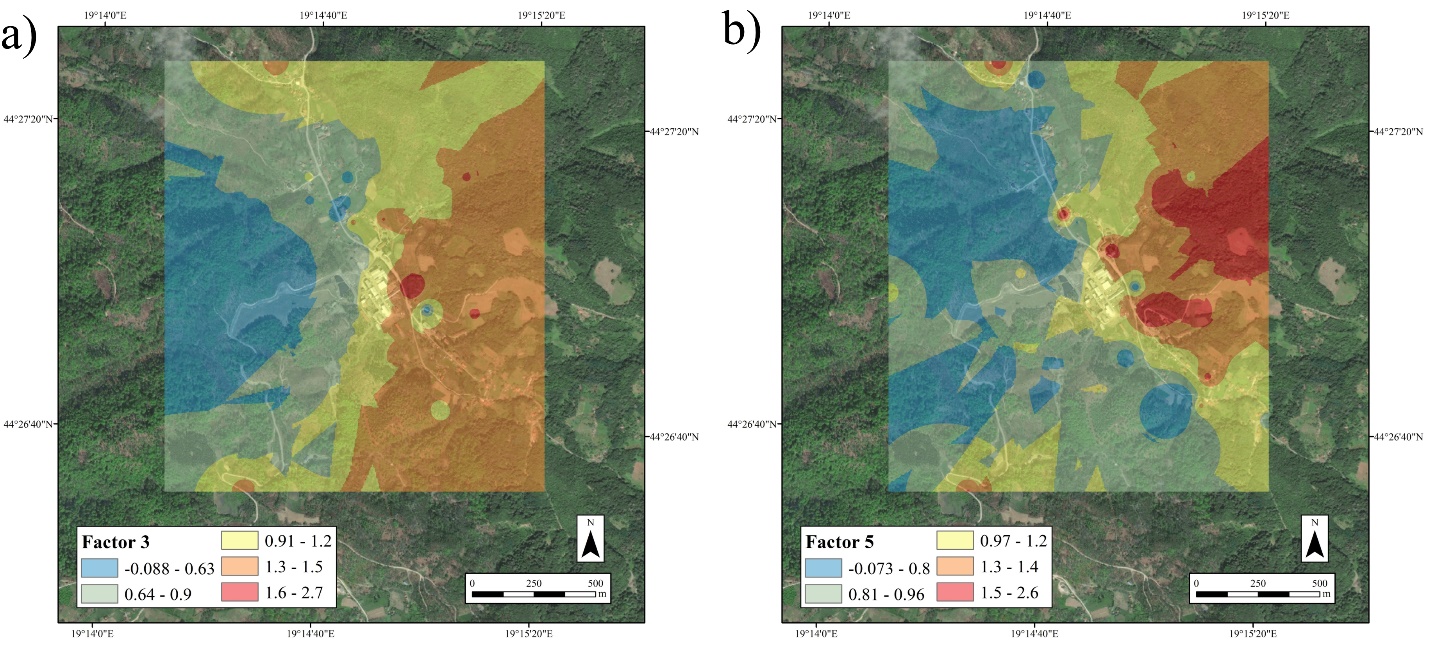


**Figure S4**. Spatial influence for PMF factors a) III factor b) V factor


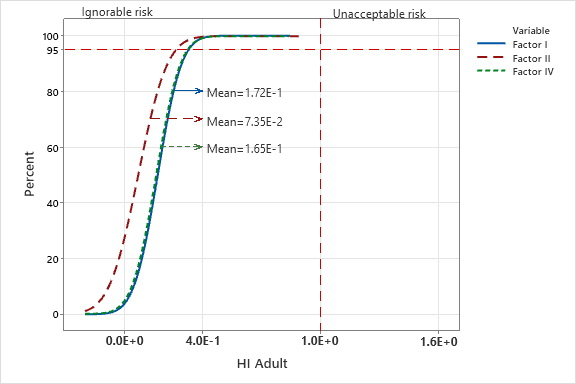


**Figure S5.** Probability distribution for non-carcinogenic health risk (HI) for adults

Reference:

1. Čakmak, D., Perović, V., Kresović, M., Pavlović, D., Pavlović, M., Mitrović, M., Pavlović, P., 2020. Sources and a health risk assessment of potentially toxic elements in dust at children’s playgrounds with artificial surfaces: A case study in Belgrade. Arch. Environ. Contam. Toxicol. 78, 190-205. <https://doi.org/10.1007/s00244-019-00702-0>
2. Jia, Z., Li, S., Wang, L., 2018. Assessment of soil heavy metals for eco-environment and human health in a rapidly urbanization area of the upper Yangtze Basin. Sci. Rep. 8, 3256. <https://doi.org/10.1038/s41598-018-21569-6>.
3. MEP, 2016. Project of the Ministry of Ecology and Protection. The state of non-agricultural land in industrial zones of larger cities in the Republic of Serbia from the aspect of biological and chemical quality. (in Serbian) <https://www.ekologija.gov.rs/sites/default/files/old-documents/Zemljiste/Projekti/Stanje-zemljista-industrijskih-zona-vecih-gradova4.pdf> (accessed on 2 October 2023).
4. U.S. Department of Energy (USDOE), 2011. The risk assessment information system (RAIS). U.S. Department of Energy’s Oak Ridge Operations Office (ORO).
5. U.S. Environmental Protection Agency (USEPA), 2020. Regional Screening Levels (RSLs)-User’s Guide. <https://www.epa.gov/risk/regional-screening-levels-rsls-users-guide>

(accessed on 2 October 2023).
